# Physicochemical attack against solid tumors based on the reversal of direction of entropy flow: an attempt to introduce thermodynamics in anticancer therapy

**DOI:** 10.1186/1746-1596-1-43

**Published:** 2006-11-15

**Authors:** Liaofu Luo, Joseph Molnar, Hui Ding, Xiaogui Lv, Gabriella Spengler

**Affiliations:** 1 Laboratory of Theoretical Biophysics, Faculty of Science and Technology, Inner Mongolia University, Hohhot, China; 2 Department of Medical Microbiology, Albert Szent-Gyorgyi Medical Center, University of Szeged, Hungary

## Abstract

**Background:**

There are many differences between healthy tissue and growing tumor tissue, including metabolic, structural and thermodynamic differences. Both structural and thermodynamic differences can be used to follow the entropy differences in cancerous and normal tissue. Entropy production is a bilinear form of the rates of irreversible processes and the corresponding "generalized forces". Entropy production due to various dissipation mechanisms based on temperature differences, chemical potential gradient, chemical affinity, viscous stress and exerted force is a promising tool for calculations relating to potential targets for tumor isolation and demarcation.

**Methods:**

The relative importance of five forms of entropy production was assessed through mathematical estimation. Using our mathematical model we demonstrated that the rate of entropy production by a cancerous cell is always higher than that of a healthy cell apart from the case of the application of external energy. Different rates of entropy production by two kinds of cells influence the direction of entropy flow between the cells. Entropy flow from a cancerous cell to a healthy cell transfers information regarding the cancerous cell and propagates its invasive action to the healthy tissues. To change the direction of entropy flow, in addition to designing certain biochemical pathways to reduce the rate of entropy production by cancerous cells, we suggest supplying external energy to the tumor area, changing the relative rate of entropy production by the two kinds of cells and leading to a higher entropy accumulation in the surrounding normal cells than in the tumorous cells.

**Conclusion:**

Through the use of mathematical models it was quantitatively demonstrated that when no external force field is applied, the rate of entropy production of cancerous cells is always higher than that of healthy cells. However, when the external energy of square wave electric pulses is applied to tissues, the rate of entropy production of normal cells may exceed that of cancerous cells. Consequently, the application of external energy to the body can reverse the direction of the entropy current. The harmful effect brought about by the entropy flow from cancerous to healthy tissue can be blocked by the reversed direction of entropy current from the irradiated normal tissue around the tumor.

## Background

Entropy production, reflecting the rate of the growth of disorder, is a thermodynamic quantity of fundamental importance for a living system: following the second law of thermodynamics, the entropy of a system always increases for any non-equilibrium system if no entropy flows outward. The entropy production *σ*_*s *_is the rate of entropy increase in unit volume. It can be proved that *σ*_*s *_contains five terms [1,2]:

1, *σ*_*s*_^(1) ^the thermal flux driven by a temperature difference;

2, *σ*_*s*_^(2) ^the diffusion current driven by a chemical potential gradient;

3, *σ*_*s*_^(3) ^the chemical reaction rate driven by a Gibbs energy decrease (affinity);

4, *σ*_*s*_^(4) ^the velocity gradient coupled with viscous stress;

5, *σ*_*s*_^(5) ^the dissipation due to the work completed by an external force field.

(see Supplementary Material 2). Non-equilibrium statistical physics affords an important clue for the understanding of the self-organization phenomena of living bodies. Prigogine proved that, in the linear range of an irreversible process in non-equilibrium thermodynamics, the entropy production always takes up a minimum if local equilibrium is assumed [3]. Since the living organism is a chemical engine in which a series of chemical reactions take place one by one in an appropriate sequence, the energy transfer in an organism in normal state is so efficient that the entropy production is minimized. This means that the minimal entropy production theorem can be generalized to healthy cells [4]. Tumorous cells are structurally less ordered than healthy cells. [5] Structural variations have been found in solid tumors [6-8]. The possible role of negative entropy in tumor growth and its relation to kinetic and genetic resistance have been discussed in the literature [9]. Structural variation and entropy production are interrelated. The related phenomena in order-disorder transitions, for example, the microvascularization [6] and syntactic structure analysis of pleural tumors [7] and chromosal alterations in the non-neoplastic bronchial mucose [8] may be studied from the aspect of entropy production. The primary aim of this study is to calculate the rate of entropy production quantitatively and compare the rates of the entropy production for normal and cancerous cell tissues. We shall prove that the rate of entropy production in normal cells is always lower than that in a cancerous cell if no external force field is applied [10].

Entropy production *σ*_*s *_and entropy flow are related to each other by the continuity equation (entropy balance equation). The entropy flow contains three terms: the convection term of entropy, the conduction term relating to the transport of heat, and the conduction term relating to the transport of matter [11]. The last term is always in the direction opposite to that of the flow of matter. The first term involves the entropy transport from a site of high entropy density to one of low entropy density that accompanies the convection movement of the biological medium. Due to the homogeneity of temperature in the human body, the heat conduction term can be neglected. Hence, mainly the first and third terms contribute to the entropy flow. By comparison of their definitions, the entropy flow is seen to be related to the information flow. In fact, the entropy flow is the carrier of the information flow (see the Supplementary Material 1). The entropy flow from a normal to a cancerous cell carries the information of the healthy cell, while the entropy flow in the opposite direction carries the harmful information on the cancerous cell. Since a cancerous cell is in a high entropy state because of its disorder structure and higher entropy production rate as compared with those of a normal cell, the entropy usually flows from cancerous to normal tissue. This induces the propagation of harmful information (the information relating to the particular bias of states in the cancerous cell; see the Supplementary Material 1) from the cancerous tissue to the surrounding healthy tissue. From the aspect of anticancer therapy, it is important to design a way to block the entropy flow or, even better, to reverse the direction of entropy propagation. The design of an approach to reversal of the direction of entropy flow is the second motivation of this study. Several ways may be suggested from a theoretical consideration of entropy production and entropy flow. The first is to reduce the rate of entropy production of cancerous cells through the design of certain biochemical pathways, for example, changing the pH gradient between the cancerous and normal cells. The second is the targeted destruction of the established tumor vasculature to reduce the transport of matter from normal to cancerous cells. The third is to apply an electric field or other external energy to cells. Our study in this article will focus on the third possibility. We shall prove that the rate of entropy production of normal cells under an appropriate electric field may exceed the rate of entropy production of cancerous cells, and therefore reverse the direction of entropy flow between the cells. This may have some therapeutic effects. Thus, entropy production considerations provide new insight into cancer therapy, with potential as a diagnostic tool.

## Theoretical Methods

To assess the difference between cancerous and normal cells from the aspect of thermodynamics, we shall calculate each term of the rate of entropy production in a cell.

1. Heat flux driven by a temperature difference

A heat flux can be expressed as the product of a cell internal energy of the *U*_*cell *_and the molecular thermal motion velocity *V*_*thermal*_; the generalized force – temperature gradient can be expressed as the temperature change *δT *in a typical length *L*. Thus the rate of entropy production in a cell can be estimated as

∫σs(1)dτ≈1T2UcellVthermalδTL     (1)
 MathType@MTEF@5@5@+=feaafiart1ev1aaatCvAUfKttLearuWrP9MDH5MBPbIqV92AaeXatLxBI9gBaebbnrfifHhDYfgasaacH8akY=wiFfYdH8Gipec8Eeeu0xXdbba9frFj0=OqFfea0dXdd9vqai=hGuQ8kuc9pgc9s8qqaq=dirpe0xb9q8qiLsFr0=vr0=vr0dc8meaabaqaciaacaGaaeqabaqabeGadaaakeaadaWdbaqaaGGaciab=n8aZnaaDaaaleaacqWGZbWCaeaacqGGOaakcqaIXaqmcqGGPaqkaaaabeqab0Gaey4kIipakiabdsgaKjab=r8a0jabgIKi7oaalaaabaGaeGymaedabaGaemivaq1aaWbaaSqabeaacqaIYaGmaaaaaOGaemyvau1aaSbaaSqaaiabdogaJjabdwgaLjabdYgaSjabdYgaSbqabaGccqWGwbGvdaWgaaWcbaGaemiDaqNaemiAaGMaemyzauMaemOCaiNaemyBa0MaemyyaeMaemiBaWgabeaakmaalaaabaGae8hTdqMaemivaqfabaGaemitaWeaaiaaxMaacaWLjaWaaeWaaeaacqaIXaqmaiaawIcacaGLPaaaaaa@565A@

where the integral is over the cell volume. For normal tissue, the temperature is homogeneous, *δT *= 0, and there is no entropy production due to a thermal flux. However, some kinds of cancer exhibit a higher temperature [9]. This gives *δT *≠ 0 (in the range of several tenths of a degree). In this case, a decrease of the temperature gradient of the tumor and an increase of the housing temperature will be beneficial in lowering the rate of entropy production of such a cancerous cell.

2. Diffusion current driven by a chemical potential gradient

Tumor cells are structurally less ordered than healthy cells. This deficiency will increase the diffusion current and therefore increase the entropy production. Cancer cells change their cytoskeleton structure. For example, various tumor cells exhibit differences in the size, shape and number of their mitochondria, which are sometimes found as large aggregates in the mainly glycolytic cells [12-14] or with alterations in mitochondrial composition, structure and function as compared with the mitochondria in normal cells[12]. We may suppose that in a cancerous cell there are many compartments which have collapsed. Normally, the diffusion is mainly located in each compartment (the subcellular location of some proteins) and diffusion between different compartments can be neglected. However, in cancerous cells the diffusion between altered compartments should also be considered. Thus the difference in the rate of entropy production between cancerous and normal cells is :

∫σs(2)dτ (cancer)−∫σs(2)dτ (normal)≈kTδμδLVthermalMcell     (2.1)
 MathType@MTEF@5@5@+=feaafiart1ev1aaatCvAUfKttLearuWrP9MDH5MBPbIqV92AaeXatLxBI9gBaebbnrfifHhDYfgasaacH8akY=wiFfYdH8Gipec8Eeeu0xXdbba9frFj0=OqFfea0dXdd9vqai=hGuQ8kuc9pgc9s8qqaq=dirpe0xb9q8qiLsFr0=vr0=vr0dc8meaabaqaciaacaGaaeqabaqabeGadaaakeaadaWdbaqaaGGaciab=n8aZnaaDaaaleaacqWGZbWCaeaacqGGOaakcqaIYaGmcqGGPaqkaaaabeqab0Gaey4kIipakiabdsgaKjabes8a0jabbccaGiabcIcaOiabbogaJjabbggaHjabb6gaUjabbogaJjabbwgaLjabbkhaYjabcMcaPiabgkHiTmaapeaabaGae83Wdm3aa0baaSqaaiabdohaZbqaaiabcIcaOiabikdaYiabcMcaPaaaaeqabeqdcqGHRiI8aOGaemizaqMae8hXdqNaeeiiaaIaeiikaGIaeeOBa4Maee4Ba8MaeeOCaiNaeeyBa0MaeeyyaeMaeeiBaWMaeiykaKIaeyisIS7aaSaaaeaacqWGRbWAaeaacqWGubavaaWaaSaaaeaacqWF0oazcqWF8oqBaeaacqWF0oazcqWGmbataaGaemOvay1aaSbaaSqaaiabdsha0jabdIgaOjabdwgaLjabdkhaYjabd2gaTjabdggaHjabdYgaSbqabaGccqWGnbqtdaWgaaWcbaGaem4yamMaemyzauMaemiBaWMaemiBaWgabeaakiaaxMaacaWLjaWaaeWaaeaacqaIYaGmcqGGUaGlcqaIXaqmaiaawIcacaGLPaaaaaa@7AB3@

where the integral is over the cell volume, *M*_*cell *_is the cell mass, *δL *is the thickness of the boundary layer and *δμ *is the difference in chemical potential between the two sides of the collapsed boundary, which is of the same order as a typical chemical potential in a cell. The parameter *k *describes the mass ratio of the boundary layer of the collapsed compartments in the whole cell. Likewise, the entropy production in a normal cell can be deduced and we have

(∫σs(2)dτ (cancer)−∫σs(2)dτ (normal))/∫σs(2)dτ (normal)∼2KR/δL     (2.2)
 MathType@MTEF@5@5@+=feaafiart1ev1aaatCvAUfKttLearuWrP9MDH5MBPbIqV92AaeXatLxBI9gBaebbnrfifHhDYfgasaacH8akY=wiFfYdH8Gipec8Eeeu0xXdbba9frFj0=OqFfea0dXdd9vqai=hGuQ8kuc9pgc9s8qqaq=dirpe0xb9q8qiLsFr0=vr0=vr0dc8meaabaqaciaacaGaaeqabaqabeGadaaakeaacqGGOaakdaWdbaqaaGGaciab=n8aZnaaDaaaleaacqWGZbWCaeaacqGGOaakcqaIYaGmcqGGPaqkaaGccqWGKbazcqWFepaDcqqGGaaiaSqabeqaniabgUIiYdGccqGGOaakcqqGJbWycqqGHbqycqqGUbGBcqqGJbWycqqGLbqzcqqGYbGCcqGGPaqkcqGHsisldaWdbaqaaiab=n8aZnaaDaaaleaacqWGZbWCaeaacqGGOaakcqaIYaGmcqGGPaqkaaaabeqab0Gaey4kIipakiabdsgaKjab=r8a0jabbccaGiabcIcaOiabb6gaUjabb+gaVjabbkhaYjabb2gaTjabbggaHjabbYgaSjabcMcaPiabcMcaPiabc+caVmaapeaabaGae83Wdm3aa0baaSqaaiabdohaZbqaaiabcIcaOiabikdaYiabcMcaPaaaaeqabeqdcqGHRiI8aOGaemizaqMae8hXdqNaeeiiaaIaeiikaGIaeeOBa4Maee4Ba8MaeeOCaiNaeeyBa0MaeeyyaeMaeeiBaWMaeiykaKIaeSipIOJaeGOmaiJaem4saSKaemOuaiLaei4la8Iae8hTdqMaemitaWKaaCzcaiaaxMaadaqadaqaaiabikdaYiabc6caUiabikdaYaGaayjkaiaawMcaaaaa@7EF5@

where *R *is the cell radius.

3. Chemical reaction rate driven by affinity, by a Gibbs energy decrease

The structural change in a cancerous cell causes a corresponding change in function. Due to the decreased mitochondrial activity, glycolysis and proteolytic and lipolytic processes become the main energy sources of a cancerous cell where the extensive loss of skeletal muscle and adipose tissue occurs. The loss of adipose tissue is due to the degradation of triglycerides, while the loss of skeletal muscle is due to increased protein degradation[15]. Tumor products such as the lipid mobilizing factor (LMF)[16] and the proteolysis inducing factor (PIF)[17] have catabolic effects on the host, but the synthesis of muscle protein also decreases. Lipid mobilization produces high energy but also a great loss of fat mass in cancer patients. The most important pathway of protein degradation involving the ATP-ubiquitin, also leads to the hypermetabolism characteristic of parasitism[15]. All proteolytic and lipolytic processes, as catabolic reactions, contribute more entropy production in cancerous than in normal cells. In the following we shall study glycolysis only and compare the entropy production of glycolysis in tumorous tissue with that of the full oxidation of glucose in normal cells.

In normal cells, the full oxidation of 1 mole of glucose will release 676 kcal/mole and produces 31 moles (or 29.5 moles in the alternative pathway) of ATP[18]. Hence, the total Gibbs free energy decrease is ∑δAδ
 MathType@MTEF@5@5@+=feaafiart1ev1aaatCvAUfKttLearuWrP9MDH5MBPbIqV92AaeXatLxBI9gBaebbnrfifHhDYfgasaacH8akY=wiFfYdH8Gipec8Eeeu0xXdbba9frFj0=OqFfea0dXdd9vqai=hGuQ8kuc9pgc9s8qqaq=dirpe0xb9q8qiLsFr0=vr0=vr0dc8meaabaqaciaacaGaaeqabaqabeGadaaakeaadaaeqbqaaiabdgeabnaaBaaaleaaiiGacqWF0oazaeqaaaqaaiab=r7aKbqab0GaeyyeIuoaaaa@3347@ (normal) = 686-7.3 × 31 × 1.7 = 301.3 kcal/mole (the sum is over reactions *δ*) in the respiratory chain of a normal cell (the factor 1.7, relating to the possible higher efficiency of ATP hydrolysis energy transformed into chemical energy in a living cell, is empirically introduced). Instead, in a cancerous cell glycolysis is the main process where, the total free energy release is 52 kcal/mole and 1 mole of glucose produces only 2 moles of ATP. Correspondingly, the Gibbs free energy decrease is ∑δAδ
 MathType@MTEF@5@5@+=feaafiart1ev1aaatCvAUfKttLearuWrP9MDH5MBPbIqV92AaeXatLxBI9gBaebbnrfifHhDYfgasaacH8akY=wiFfYdH8Gipec8Eeeu0xXdbba9frFj0=OqFfea0dXdd9vqai=hGuQ8kuc9pgc9s8qqaq=dirpe0xb9q8qiLsFr0=vr0=vr0dc8meaabaqaciaacaGaaeqabaqabeGadaaakeaadaaeqbqaaiabdgeabnaaBaaaleaaiiGacqWF0oazaeqaaaqaaiab=r7aKbqab0GaeyyeIuoaaaa@3347@ (cancer) = 52-7.3 × 2 = 37.4 kcal/mole in a cancerous cell. On the other hand, it was demonstrated through positron emission tomography scanning that tumor cells absorb more glucose than normal cells. Cancer cells metabolise glucose at a rate of approximately 20 times that of normal tissue [19]. For example, while normal cells take up 2~16 g glucose from 1000 ml blood, the cancerous cells with the same tissue origin will take 70 g. Thus, the glucose consumption in cancerous cells is much higher than in healthy cells. Simultaneously, no matter whether the oxygen supply in cells is enough or not, the tumor always maintains an efficiency of glycolysis 70–80 times higher than normal. From the comparison of the ATP molecule number produced in glycolysis and glucose oxidation in the respiratory chain, we estimate the average reaction rate of the oxidation of glucose in unit volume of a cancerous cell (*J*_*c*_) to be at least 15~20 times higher than that in a normal cell (*J*_*n*_). Thus, the rates of entropy production in a cell are

∫σs(3)dτ=301.31T∫Jndτ     (normal)∫σs(3)dτ=37.41T∫Jcdτ≥(561∼748)×1T∫Jndτ     (cancer)     (3)
 MathType@MTEF@5@5@+=feaafiart1ev1aaatCvAUfKttLearuWrP9MDH5MBPbIqV92AaeXatLxBI9gBaebbnrfifHhDYfgasaacH8akY=wiFfYdH8Gipec8Eeeu0xXdbba9frFj0=OqFfea0dXdd9vqai=hGuQ8kuc9pgc9s8qqaq=dirpe0xb9q8qiLsFr0=vr0=vr0dc8meaabaqaciaacaGaaeqabaqabeGadaaakeaafaqaaeGabaaabaWaa8qaaeaaiiGacqWFdpWCdaqhaaWcbaGaem4CamhabaGaeiikaGIaeG4mamJaeiykaKcaaaqabeqaniabgUIiYdGccqWGKbazcqWFepaDcqGH9aqpcqaIZaWmcqaIWaamcqaIXaqmcqGGUaGlcqaIZaWmdaWcaaqaaiabigdaXaqaaiabdsfaubaadaWdbaqaaiabdQeaknaaBaaaleaacqWGUbGBaeqaaaqabeqaniabgUIiYdGccqWGKbazcqWFepaDcaWLjaGaaCzcaiabcIcaOiabb6gaUjabb+gaVjabbkhaYjabb2gaTjabbggaHjabbYgaSjabcMcaPaqaamaapeaabaGae83Wdm3aa0baaSqaaiabdohaZbqaaiabcIcaOiabiodaZiabcMcaPaaaaeqabeqdcqGHRiI8aOGaemizaqMae8hXdqNaeyypa0JaeG4mamJaeG4naCJaeiOla4IaeGinaqZaaSaaaeaacqaIXaqmaeaacqWGubavaaWaa8qaaeaacqWGkbGsdaWgaaWcbaGaem4yamgabeaaaeqabeqdcqGHRiI8aOGaemizaqMae8hXdqNaeyyzImRaeiikaGIaeGynauJaeGOnayJaeGymaeJaeSipIOJaeG4naCJaeGinaqJaeGioaGJaeiykaKIaey41aq7aaSaaaeaacqaIXaqmaeaacqWGubavaaWaa8qaaeaacqWGkbGsdaWgaaWcbaGaemOBa4gabeaaaeqabeqdcqGHRiI8aOGaemizaqMae8hXdqNaaCzcaiaaxMaacqGGOaakcqqGJbWycqqGHbqycqqGUbGBcqqGJbWycqqGLbqzcqqGYbGCcqGGPaqkaaGaaCzcaiaaxMaadaqadaqaaiabiodaZaGaayjkaiaawMcaaaaa@9177@

The rate of entropy production in the carbon energy source reaction of a cancerous cell is estimated to be 2 or more times higher than that in a healthy cell. Furthermore, if the strong proteolytic and lipolytic processes in cancer are taken into account, the entropy production of a cancerous cell will be even higher.

4. Velocity gradient coupling with viscous stress

The difference in viscous stress (inner friction in a cellular fluid) – induced entropy production between a cancerous and a normal cell stems mainly from the contribution in the boundary layers of the compartments. The stress is related to medium deformation by a linear law through viscosity coefficient *η*. Thus:

∫σs(4)dτ (cancer)−∫σs(4)dτ (normal)≈ηT〈(∂iVj)2〉B∫boundarydτ     (4.1)
 MathType@MTEF@5@5@+=feaafiart1ev1aaatCvAUfKttLearuWrP9MDH5MBPbIqV92AaeXatLxBI9gBaebbnrfifHhDYfgasaacH8akY=wiFfYdH8Gipec8Eeeu0xXdbba9frFj0=OqFfea0dXdd9vqai=hGuQ8kuc9pgc9s8qqaq=dirpe0xb9q8qiLsFr0=vr0=vr0dc8meaabaqaciaacaGaaeqabaqabeGadaaakeaadaWdbaqaaGGaciab=n8aZnaaDaaaleaacqWGZbWCaeaacqGGOaakcqaI0aancqGGPaqkaaaabeqab0Gaey4kIipakiabdsgaKjab=r8a0jabbccaGiabcIcaOiabbogaJjabbggaHjabb6gaUjabbogaJjabbwgaLjabbkhaYjabcMcaPiabgkHiTmaapeaabaGae83Wdm3aa0baaSqaaiabdohaZbqaaiabcIcaOiabisda0iabcMcaPaaaaeqabeqdcqGHRiI8aOGaemizaqMae8hXdqNaeeiiaaIaeiikaGIaeeOBa4Maee4Ba8MaeeOCaiNaeeyBa0MaeeyyaeMaeeiBaWMaeiykaKIaeyisIS7aaSaaaeaacqWF3oaAaeaacqWGubavaaWaaaWabeaacqGGOaakcqGHciITdaWgaaWcbaGaemyAaKgabeaakiabdAfawnaaBaaaleaacqWGQbGAaeqaaOGaeiykaKYaaWbaaSqabeaacqaIYaGmaaaakiaawMYicaGLQmcadaWgaaWcbaGaeeOqaieabeaakmaapefabaGaemizaqMae8hXdqhaleaacqqGIbGycqqGVbWBcqqG1bqDcqqGUbGBcqqGKbazcqqGHbqycqqGYbGCcqqG5bqEaeqaniabgUIiYdGccaWLjaGaaCzcamaabmaabaGaeGinaqJaeiOla4IaeGymaedacaGLOaGaayzkaaaaaa@7F30@

where *V*_*j *_is the fluid component velocity parallel to the boundary and *∂*_*i*_*V*_*j *_is the variation in the direction perpendicular to it; ⟨ ⟩_B _indicates the average over the boundary layer. Likewise, the entropy production in a normal cell can be estimated. We obtain

(∫σs(4)dτ (cancer)−∫σs(4)dτ (normal))/∫σs(4)dτ (normal)≈k〈(∂iVj)2〉B〈(∂iVj)2〉V     (4.2)
 MathType@MTEF@5@5@+=feaafiart1ev1aaatCvAUfKttLearuWrP9MDH5MBPbIqV92AaeXatLxBI9gBaebbnrfifHhDYfgasaacH8akY=wiFfYdH8Gipec8Eeeu0xXdbba9frFj0=OqFfea0dXdd9vqai=hGuQ8kuc9pgc9s8qqaq=dirpe0xb9q8qiLsFr0=vr0=vr0dc8meaabaqaciaacaGaaeqabaqabeGadaaakeaacqGGOaakdaWdbaqaaGGaciab=n8aZnaaDaaaleaacqWGZbWCaeaacqGGOaakcqaI0aancqGGPaqkaaaabeqab0Gaey4kIipakiabdsgaKjab=r8a0jabbccaGiabcIcaOiabbogaJjabbggaHjabb6gaUjabbogaJjabbwgaLjabbkhaYjabcMcaPiabgkHiTmaapeaabaGae83Wdm3aa0baaSqaaiabdohaZbqaaiabcIcaOiabisda0iabcMcaPaaaaeqabeqdcqGHRiI8aOGaemizaqMae8hXdqNaeeiiaaIaeiikaGIaeeOBa4Maee4Ba8MaeeOCaiNaeeyBa0MaeeyyaeMaeeiBaWMaeiykaKIaeiykaKIaei4la8Yaa8qaaeaacqWFdpWCdaqhaaWcbaGaem4CamhabaGaeiikaGIaeGinaqJaeiykaKcaaaqabeqaniabgUIiYdGccqWGKbazcqWFepaDcqqGGaaicqGGOaakcqqGUbGBcqqGVbWBcqqGYbGCcqqGTbqBcqqGHbqycqqGSbaBcqGGPaqkcqGHijYUcqWGRbWAdaWcaaqaamaaamqabaWaaeWaaeaacqGHciITdaWgaaWcbaGaemyAaKgabeaakiabdAfawnaaBaaaleaacqWGQbGAaeqaaaGccaGLOaGaayzkaaWaaWbaaSqabeaacqaIYaGmaaaakiaawMYicaGLQmcadaWgaaWcbaGaemOqaieabeaaaOqaamaaamqabaWaaeWaaeaacqGHciITdaWgaaWcbaGaemyAaKgabeaakiabdAfawnaaBaaaleaacqWGQbGAaeqaaaGccaGLOaGaayzkaaWaaWbaaSqabeaacqaIYaGmaaaakiaawMYicaGLQmcadaWgaaWcbaGaemOvayfabeaaaaGccaWLjaGaaCzcamaabmaabaGaeGinaqJaeiOla4IaeGOmaidacaGLOaGaayzkaaaaaa@9134@

where ⟨ ⟩_V _denotes the average over the total cell.

5. Dissipation due to work performed by an external force field acting on a biological medium Tumorous cells exhibit dielectric characteristics that are distinct from those of healthy cells. Higher dielectric permittivity and specific electroconductivity have been measured in cancerous cells[20]. This means that, although tumorous cells are structurally less ordered than healthy cells, due to the reduced subcellular compartmentalization, they are dielectrically more spatially ordered. The formation of dielectric zones accompanying membrane depolarization in cancerous cells has been observed[9]. A particular dielectric region may be characterized by the controlled movement of charged particles or the orientation of polar molecules in an electric field[21]. Accordingly, we expect different responses of cancerous and normal cells, and therefore different rates of entropy production in an applied electric field. Changes in electrical potential may result in dramatic changes in cellular functions and structures. To calculate the entropy production σs(5)
 MathType@MTEF@5@5@+=feaafiart1ev1aaatCvAUfKttLearuWrP9MDH5MBPbIqV92AaeXatLxBI9gBaebbnrfifHhDYfgasaacH8akY=wiFfYdH8Gipec8Eeeu0xXdbba9frFj0=OqFfea0dXdd9vqai=hGuQ8kuc9pgc9s8qqaq=dirpe0xb9q8qiLsFr0=vr0=vr0dc8meaabaqaciaacaGaaeqabaqabeGadaaakeaaiiGacqWFdpWCdaqhaaWcbaGaem4CamhabaGaeiikaGIaeGynauJaeiykaKcaaaaa@32BC@, let us consider a simplified model. Let us suppose a homogeneous electrostatic field (***E***) and the polar molecules in a cell approximated as a set of electric dipoles (***d***_***i***_, *i *= 1,...*N*) with equal dipole moments but oriented differently. No work will be done by the electric field if only the movement of the center of mass of the dipole is considered. However, as the angle *θ *between ***E ***and ***d***_***i ***_is changed, the work performed by the electric field will be *dW *= -*Ed*_*i *_sin *θdθ*. Its average over direction is *Ed*_*i*_. Thus, for a normal cell the total work completed by the electric field is *NEd *(|**d**_**i**_| = *d*). However, for a cancerous cell, *N *dipoles are grouped into *s *sets. In each set, *m *(= *N/s*) dipoles are summed coherently. The total work performed by the electric field will be less than normal by a factor sN
 MathType@MTEF@5@5@+=feaafiart1ev1aaatCvAUfKttLearuWrP9MDH5MBPbIqV92AaeXatLxBI9gBaebbnrfifHhDYfgasaacH8akY=wiFfYdH8Gipec8Eeeu0xXdbba9frFj0=OqFfea0dXdd9vqai=hGuQ8kuc9pgc9s8qqaq=dirpe0xb9q8qiLsFr0=vr0=vr0dc8meaabaqaciaacaGaaeqabaqabeGadaaakeaadaGcaaqaamaalaaabaGaem4CamhabaGaemOta4eaaaWcbeaaaaa@2F6B@. Since the difference in dielectric permittivity between cancerous and healthy cells is very marked[20], the number *m *= *N/s *should not be small. Therefore, an electric (static) field applied to the body will moderate the entropy production effect of cancerous as compared with normal cells. On the other hand, the time needed for dipole orientation in an electric field is very short. The numerical estimation gives a dipole orientation time of the order of 10^-11 ^s. Hence, we may introduce square wave electric pulses (SWEP) instead of an electrostatic field; that is, the electrostatic field continuously switched on and off many times in a given time duration, to increase the entropy production. (The width of the SWEP is assumed to be larger than the dipole orientation time.) Setting the pulse frequency *ν *of the SWEP we have the rate of entropy production in a cell due to the applied electric field

∫σs(5)dτ=ν NEd/T     (normal)∫σs(5)dτ=ν Ns Ed/T     (cancer)     (5)
 MathType@MTEF@5@5@+=feaafiart1ev1aaatCvAUfKttLearuWrP9MDH5MBPbIqV92AaeXatLxBI9gBaebbnrfifHhDYfgasaacH8akY=wiFfYdH8Gipec8Eeeu0xXdbba9frFj0=OqFfea0dXdd9vqai=hGuQ8kuc9pgc9s8qqaq=dirpe0xb9q8qiLsFr0=vr0=vr0dc8meaabaqaciaacaGaaeqabaqabeGadaaakeaafaqaaeGabaaabaWaa8qaaeaaiiGacqWFdpWCdaqhaaWcbaGaee4CamhabaGaeeikaGIaeeynauJaeeykaKcaaaqabeqaniabgUIiYdGccqWGKbazcqWFepaDcqGH9aqpcqWF9oGBcqqGGaaicqWGobGtcqWGfbqrcqWGKbazcqGGVaWlcqWGubavcaWLjaGaaCzcaiabcIcaOiabb6gaUjabb+gaVjabbkhaYjabb2gaTjabbggaHjabbYgaSjabcMcaPaqaamaapeaabaGae83Wdm3aa0baaSqaaiabbohaZbqaaiabbIcaOiabbwda1iabbMcaPaaaaeqabeqdcqGHRiI8aOGaemizaqMae8hXdqNaeyypa0Jae8xVd4MaeeiiaaYaaOaaaeaacqWGobGtcqWGZbWCaSqabaGccqqGGaaicqWGfbqrcqWGKbazcqGGVaWlcqWGubavcaWLjaGaaCzcamaabmaabaGaee4yamMaeeyyaeMaeeOBa4Maee4yamMaeeyzauMaeeOCaihacaGLOaGaayzkaaaaaiaaxMaacaWLjaWaaeWaaeaacqaI1aqnaiaawIcacaGLPaaaaaa@712D@

## Results

The mathematical estimations of five forms of entropy production are presented in Table 1. We find that:

**Table 1:** Rates of entropy production in normal and cancerous cells

	∫σs(i)dτ MathType@MTEF@5@5@+=feaafiart1ev1aaatCvAUfKttLearuWrP9MDH5MBPbIqV92AaeXatLxBI9gBaebbnrfifHhDYfgasaacH8akY=wiFfYdH8Gipec8Eeeu0xXdbba9frFj0=OqFfea0dXdd9vqai=hGuQ8kuc9pgc9s8qqaq=dirpe0xb9q8qiLsFr0=vr0=vr0dc8meaabaqaciaacaGaaeqabaqabeGadaaakeaadaWdbaqaaGGaciab=n8aZnaaDaaaleaacqqGZbWCaeaacqqGOaakieGacqGFPbqAcqqGPaqkaaaabeqab0Gaey4kIipakiabdsgaKjab=r8a0baa@382C@ (normal)	∫σs(i)dτ MathType@MTEF@5@5@+=feaafiart1ev1aaatCvAUfKttLearuWrP9MDH5MBPbIqV92AaeXatLxBI9gBaebbnrfifHhDYfgasaacH8akY=wiFfYdH8Gipec8Eeeu0xXdbba9frFj0=OqFfea0dXdd9vqai=hGuQ8kuc9pgc9s8qqaq=dirpe0xb9q8qiLsFr0=vr0=vr0dc8meaabaqaciaacaGaaeqabaqabeGadaaakeaadaWdbaqaaGGaciab=n8aZnaaDaaaleaacqqGZbWCaeaacqqGOaakieGacqGFPbqAcqqGPaqkaaaabeqab0Gaey4kIipakiabdsgaKjab=r8a0baa@382C@ (cancer)	Cancer/Normal	Parameter choice (in CGS unit)
***i *= 1**	0	1.1 × 10^-25^		*T *= 310, *δT *= 0.4, *U*_cell _= 0.13 × 10^-20^, *V*_thermal _= 10^-1^, L = 0.5 × 10^-2^
***i *= 2**	0.42 × 10^-23^	0.47 × 10^-23^	1.1	*T *= 310, *M*_cell _= 0.65 × 10^-7^, *μ*_ATP _= 10^-15^, 2*R *= 0.5 × 10^-2^, *V*_thermal _= 10^-1^, *δL *= 0.5 × 10^-4^, *k *= 10^-3^
***i *= 3**	0.43 × 10^-8^	1 × 10^-7^	2	*A*_normal _= 301.3 × 0.695 × 10^-13 ^∫J_*n*_*dν *= 0.63 × 10^5^, *A*_cancer _= 37.4 × 0.695 × 10^-13 ^∫J_*c*_*dν *>15 ∫J_*n*_*dν*
***i *= 4**	0.13 × 10^-10^	0.15 × 10^-10^	1.1	*T *= 310, *η *= 0.015, *V*_cell _= 0.65 × 10^-7^, *k *= 10^-3^, (∂iVj)ν=10−20.5×10−2 MathType@MTEF@5@5@+=feaafiart1ev1aaatCvAUfKttLearuWrP9MDH5MBPbIqV92AaeXatLxBI9gBaebbnrfifHhDYfgasaacH8akY=wiFfYdH8Gipec8Eeeu0xXdbba9frFj0=OqFfea0dXdd9vqai=hGuQ8kuc9pgc9s8qqaq=dirpe0xb9q8qiLsFr0=vr0=vr0dc8meaabaqaciaacaGaaeqabaqabeGadaaakeaacqGGOaakcqGHciITdaWgaaWcbaGaemyAaKgabeaakiabdAfawnaaBaaaleaacqWGQbGAaeqaaOGaeiykaKYaaSbaaSqaaGGaciab=17aUbqabaGccqGH9aqpdaWcaaqaaiabigdaXiabicdaWmaaCaaaleqabaGaeyOeI0IaeGOmaidaaaGcbaGaeGimaaJaeiOla4IaeGynauJaey41aqRaeGymaeJaeGimaaZaaWbaaSqabeaacqGHsislcqaIYaGmaaaaaaaa@43E7@(∂iVj)B=10−20.5×10−3 MathType@MTEF@5@5@+=feaafiart1ev1aaatCvAUfKttLearuWrP9MDH5MBPbIqV92AaeXatLxBI9gBaebbnrfifHhDYfgasaacH8akY=wiFfYdH8Gipec8Eeeu0xXdbba9frFj0=OqFfea0dXdd9vqai=hGuQ8kuc9pgc9s8qqaq=dirpe0xb9q8qiLsFr0=vr0=vr0dc8meaabaqaciaacaGaaeqabaqabeGadaaakeaacqGGOaakcqGHciITdaWgaaWcbaGaemyAaKgabeaakiabdAfawnaaBaaaleaacqWGQbGAaeqaaOGaeiykaKYaaSbaaSqaaGqaciab=jeacbqabaGccqGH9aqpdaWcaaqaaiabigdaXiabicdaWmaaCaaaleqabaGaeyOeI0IaeGOmaidaaaGcbaGaeGimaaJaeiOla4IaeGynauJaey41aqRaeGymaeJaeGimaaZaaWbaaSqabeaacqGHsislcqaIZaWmaaaaaaaa@433E@
***i *= 5 **(SWEP)	0.16 × 10^-9^	0.05 × 10^-9^	0.33	*E *= 3 × 10^3 ^volt/cm, *ν *= 100 *d *= 5 × 10^-17^, *N *= 10^6^, *s/N *= 0.1, *T *= 310

Five kinds of entropy production rate in normal and cancerous cells are given in the Table. Their ratios are listed in the fourth column. Parameter definitions are to be found in the text. All parameters are given in CGS units except those specified.

1. The rate of entropy production for a cell without external energy input is described by the first four contributions (*i *= 1,2,3,4), in which the rates due to chemical reaction and viscous stress (*i *= 3,4) are much higher than other two terms (*i *= 1,2); the rate of entropy production in a cancerous cell is generally higher than that in a normal cell. Although there is some arbitrariness in parameter choice, the above conclusion generally holds independently of the choice. The result is consistent with the point of the minimal entropy production theorem.

2. The entropy production for a normal cell due to an external force field (an electrostatic field or SWEP) is generally higher than that for a cancerous cell under the assumption of different dielectric property of cancer. In the SWEP case, the magnitude of the rate of production can be adjusted through change of the pulse frequency as well as the field strength. With an appropriate strength and frequency, the total entropy production for a normal cell may exceed that for cancer cell and reverse the direction of entropy flow between these two kinds of cells.

## Discussion

1. Due to the higher entropy in cancer, the convection of entropy proceeds in the direction from cancerous to healthy cells. Due to the higher temperature that a cancer cell may have, the conduction of entropy related to heat transport also proceeds in the direction from cancerous to healthy cells. The third term of entropy flow, the conduction of entropy related to the transport of matter, is in the opposite direction to the matter transport. If the flow of matter transport is mainly from healthy to cancerous cells, then the heat conduction is again in the direction from cancerous to healthy cells. Thus, entropy generally flows from cancerous cells to healthy ones if no special therapeutic design has been introduced. Entropy accumulation in a cell is a harmful factor. The higher rate of entropy production in cancerous cells and its flow to the surrounding normal cells causes toxic action on normal cells (see Additional file 1). The toxic effect is proportional to the average entropy gain of normal cells from cancerous cells. Accordingly, it becomes stronger and stronger as the population of normal cells decreases and the population of cancerous cells increases.

2. Among the five forms of entropy production, the chemical reaction is of special importance. In the above discussion only the glucose metabolism has been taken into account. The total rate of entropy production from all kinds of chemical reaction will be much higher. If we consider the higher rate of entropy production in the main energy source reaction of a cancerous cell, how to modify the glucose metabolism pathway and lower rate of entropy production is a great problem in cancer therapy. In studies of entropy production due to chemical reactions, the affinity *A*_*δ *_is usually calculated from the standard free energy change (at pH = 7). Although most biochemical reactions take place at or near pH 7, nonstandard conditions may occur in a living cell. In this case, there will be a correction to *A*_*δ*_. One may design an approach to change the acidity of tumorouscells by producing an additional pH gradientbetween cancerous and normal cells. This will change the relative magnitude of the rate of entropy production, and in turn reverse the direction of entropy flow between these two kinds of cells.

3. An important approach to the change of entropy flow is to reduce the transport of matter from normal to cancerous cells. Angiogenesis as a therapeutic target has recently been widely discussed. It has become apparent that the targeted destruction of the established tumour vasculature is an avenue leading to exciting therapeutic opportunities[22]. From our point of view, the modulation of angiogenesis and the lowering of the glucose supply to the cancerous cells are favourable for decreasing entropy production and reversing the direction of entropy flow.

4. An increasing of the housing temperature can reverse the direction of entropy flow. Simultaneously, it lowers the temperature gradient in tumorous cells and reduces the rate of their entropy production that comes from the heat flux. Both factors are of benefit in cancer therapy. However, mathematical estimation indicates, that the entropy production due to heat is only a very small fraction of the total entropy production. This explains why the effectiveness of hyperthermiaused as therapy is very low. Another factor which should be considered is that the heat tolerance of normal cells is less than that of cancerous cells. The heat-induced entropy increase is more harmful for healthy cells than for tumorous cells.

5. We have demonstrated that if no external field is applied, the rate of entropy production of cancerous cells is always higher than that of healthy cells. However, when an external force field is applied, the rate of entropy production of normal cells may exceed that of cancerous cells. This provides a very effective approach to the change of the direction of entropy flow. By calculation of the work performed by a static electric field, we have shown that its contribution to entropy production is lower for cancerous than for normal cells. To enlarge the effect, we can use SWEP, which is equivalent to switching the electric field on and off continuously. For pulse frequency *ν*, the rate of entropy production increases *ν *times. On the use of SWEP of higher frequency, the field-induced entropy production of a cell would be comparable to the chemical reaction-induced entropy production. Thus, an applied electric field with sufficient intensity and frequency may effectively reduce the entropy production difference between normal and cancerous cells. (We have also investigated the effect of ultrasound absorption in cells and proved that the ultrasound dissipation in a normal cell may be greater than that in a cancerous cell, and the direction of entropy flow can therefore be reversed. Data are not shown here.)Therefore, the external energy (an electric field or ultrasound) may effectively alter the direction of entropy current between cells and remove the harmful information transfer from cancerous to healthy cells. The locally applied strong electric field which destabilizes cell membranes in the presence of a drug has been used in electrochemotherapy[23] and has proved effective in skin cancer[24]. Recently, combined electric field (SWEP) and ultrasound therapy was reported as a novel antitumor treatment[25]. Based on the theoretical investigation of entropy production and entropy flow, our proposal on SWEP (possibly combined with ultrasound) may afford new insight into cancer therapy.

## Conclusion

Through the use of general theory of non-equilibrium thermodynamics and simplified model of cells the five terms of entropy production due to various dissipation mechanisms, namely, entropy productions due to temperature differences, chemical potential gradient, chemical affinity, viscous stress and exerted force are quantitatively calculated for healthy and cancerous cells respectively. It was demonstrated that when no external force field is applied, the rate of entropy production of cancerous cells is always higher than that of healthy cells. However, when the external energy of an electrostatic field or equivalently, a square wave electric pulses is applied to tissues, the rate of entropy production of normal cells may exceed that of cancerous cells. Consequently, the application of external energy to the body can reverse the direction of the entropy current. Because of entropy flow is the carrier of the information flow the entropy flow from a normal to a cancerous cell carries the information of the healthy cell, while the entropy flow in the opposite direction carries the harmful information on the cancerous cell. Therefore, it is expected that the harmful effect brought about by the entropy flow from cancerous to healthy tissue can be blocked by the reversal of direction of entropy current under the applied electric field (SWEP) interaction. This provides a new insight into cancer therapy, with potential as a diagnostic tool.

## Competing interests

The author(s) declare that they have no competing interests.

## Authors' contributions

LL calculated the entropy production for cancer and normal cell respectively, proposed the direction of entropy flow as carrier of information flow can be reversed by an applied elctric field, and wrote the manuscript. JM summarized the biological difference between two kinds of cells, conceived the thermodynamic approach to anticancer and participated in the writing of the manuscript. HD collected the related data of cells and carried out a part of calculation. XL estimated the entropy production due to some generalized forces. GS participated in the writing of the manuscript and performed the work relating to the online submission on the manuscript. All authors have read and approved the final manuscript.

## Supplementary Material

Additional File 1 Supplementary Material. The data provided give brief discussions on: 1. Relation between information quantity and thermodynamic entropy; 2. General theory on entropy production. Click here for file
