# Supplementary material for: Physicochemical attack against solid tumors based on the reversal of direction of entropy flow: an attempt to introduce thermodynamics in anticancer therapy
Source: Diagn Pathol. 2006 Nov 15;1:43. doi: 10.1186/1746-1596-1-43 (PMC1685662; doi:10.1186/1746-1596-1-43)
Supplement: Additional File 1 — Supplementary Material. The data provided give brief discussions on: 1. Relation between information quantity and thermodynamic entropy; 2. General theory on entropy production. [file 1746-1596-1-43-S1.doc]

**Physicochemical attack against solid tumors based on the**

**reversal of direction of entropy flow：an attempt to**

**introduce thermodynamics in anticancer therapy**

Liaofu Luo^1*^， Joseph Molnar^2*^， Hui Ding^1^，Xiaogui Lv^1^，Gabriella Spengler^2^

^1^ Laboratory of Theoretical Biophysics, Faculty of Science and Technology, Inner Mongolia University, Hohhot, China

^2^ Department of Medical Microbiology, Albert Szent-Gyorgyi Medical Center, University of Szeged, Hungary

* Corresponding author

Email addresses

LL: [lfluo@mail.imu.edu.cn](mailto:lfluo@mail.imu.edu.cn)

JM: [molnarj@comser.szote.u-szeged.hu](mailto:molnarj@comser.szote.u-szeged.hu)

**Supplementary Material**

1. **Relation between information quantity and thermodynamic entropy**

Thermodynamic entropy is expressed by

$S=k_{B}\ln W$ (S1)

where *W* is the number of microscopic states relating to a given macroscopic thermodynamic state and *k_B_* is the Boltzmann constant. Entropy is a measure of disorder. From general physical principles, SchrÖdinger first indicated that life should remain in a low-entropy state [1] and this point has been widely accepted by scientists. To clarify the Shannon information quantity, let us consider the information conveyed by the symbols *s_i_* of a source *S* {*s_i_*}, the probability of *s_i_* being *p_i_* . The information quantity represents how much information is gained by knowing that *S* has definitely emitted the *i*-th symbol *s_i_*; this also represents our prior uncertainty as to whether *s_i_* will be emitted, and our surprise on learning that it has been emitted. Thus, the concept of information quantity is essentially similar to the description of entropy, which explains why we usually refer to the information quantity as information entropy. Mathematically, for a system with a given distribution of probable states, the Shannon information quantity is defined by

$I=-\sum_{i} p_{i}\log_{2}p_{i}$ (S2)

where *p_i_* is the probability of occurrence of the *i*-th state. For an equiprobable distribution of *N* states, $p_{i}=\frac{1}{N}$ and we have

$I=\log_{2}N=\frac{1}{\ln2}\ln N$ （S3）

This gives the information quantity *I* proportional to the thermodynamic entropy *S*. It can be proved that proportionality exists between the information quantity and entropy even for a non-equiprobable distribution of states. More rigorously, since the number of microscopic states *W* is a very large number while the number of states *N* in the definition of the Shannon information quantity is generally much smaller than *W*, we should say that the information quantity is the projection of thermodynamic entropy in microscopic phase-space to the subspace spanned by *N* macroscopic states [2].

For example, the thermodynamic entropy of a cancerous cell is different from that of a normal cell due to the more disordered structure of the cancerous cell. Correspondingly, the information inherent in a cancerous cell is different from that in a normal cell. The information quantities in cancerous and normal cells are both described by equation (S2), but they have different distributions of {*p_i_*} (*p_i_* is the probability of the *i*-th chemical, morphological, structural or physiological state of the cell), *p_i_*(cancer) ≠ *p_i_* (normal) (*i* = 1,…,*N*). We describe the information relating to a particular set of {*p_i_*} in a cancerous cell, {*p_i_*(cancer)}, as harmful information, which reflects the particular bias of the states in a tumor. The term refers to the information or the distribution {*p_i_*(cancer)} in a cancerous cell that deviates from the normal value. Similarly, the information on a healthy cell is defined by the particular set of {*p_i_*} in a healthy cell, {*p_i_*(normal)}.

The entropy of a system (a normal cell, a cancerous cell, etc.) changes with time, obeying the continuity equation (entropy balance equation) [3]:

$\begin{matrix} \frac{dS}{dt}=\int\sigma_{s}d\tau+\text{(net rate of entropy flow through boundary)} \\ \text{=}\int\sigma_{s}d\tau+(\text{entropy flow rate in})-(\text{entropy flow rate out}) \end{matrix}$ (S4)

where *σ*_s_ (called entropy production) is the rate of entropy production in unit volume. Following the second law of thermodynamics, the entropy production is always positive. Only when the entropy production is canceled by the outward entropy flow can the system remain in an ordered low-entropy state. The entropy flow consists of three parts: the convection term of entropy, the conduction term relating to the transport of heat, and the conduction term relating to the transport of matter. The last term is always in the direction opposite to the diffusion flow of matter. The first term involves the entropy transport from a site of high entropy density to one of low entropy density that accompanies convection movement. Due to the homogeneity of temperature in the human body, the heat conduction term can be neglected. Hence, mainly the first and third terms contribute to the entropy flow.

From a comparison of the definitions of thermodynamic entropy and information quantity, it is easy to understand the information flow relating to entropy flow. Since the information quantity is a projection of the thermodynamic entropy, the entropy flow should be the carrier of the information flow. Thus, the entropy flow from a normal to a cancerous cell carries the information on the healthy cell, while the entropy flow in the opposite direction carries the harmful information on the cancerous cell.

1. **General theory on entropy production**

Entropy production is a thermodynamic quantity of fundamental importance for a living system since, following the second law of thermodynamics, entropy always increases for any non-equilibrium system. The entropy production *σ*_s_ is the rate of entropy increase in unit volume. It can be proved that *σ*_s_ contains five terms [4,5]:

1, *σ*_s_ ^(1)^ the thermal flux driven by a temperature difference;

2, *σ*_s_ ^(2)^  the diffusion current driven by a chemical potential gradient;

3, *σ*_s_ ^(3)^ the chemical reaction rate driven by a Gibbs energy decrease (affinity);

4, *σ*_s_ ^(4)^  the velocity gradient coupled with viscous stress;

5, *σ*_s_ ^(5)^ the dissipation due to the work completed by an external force field.

The entropy production rate

$$\sigma_{s}=\sum_{i} \sigma_{s}^{(i)}$$

＝$\mathbf{j}_{\mathbf{q}}\cdot\nabla\frac{1}{T}$－$\sum_{\gamma} \mathbf{j}_{\mathbf{\gamma}}\cdot\nabla\frac{\mu_{\gamma}}{T}$＋ ＋$\frac{1}{T}\sum_{\gamma} \mathbf{j}_{\mathbf{\gamma}}\cdot\mathbf{F}_{\mathbf{\gamma}}$ (S5)

$A_{\delta}=-\sum_{\gamma} m_{\gamma\delta}\mu_{\gamma}$， $\mathbf{j}_{\mathbf{\gamma}}=\rho_{\gamma}(\mathbf{v}_{\mathbf{\gamma}}-\mathbf{V})$， $\mathbf{V}=\sum_{\gamma} \frac{\rho_{\gamma}\mathbf{v}_{\mathbf{\gamma}}}{\rho}$ (S6)

where **j_q_** is the heat flux, $\mathbf{j}_{\gamma}$ is the diffusion flow of component γ, ρ_γ_  is its concentration and μ_γ_ is its chemical potential, $J_{\delta}$ is the number of the *δ*-th chemical reaction in unit volume and unit time, and $A_{\delta}$ is the affinity of the *δ*-th chemical reaction, ***V*** denotes the center of mass velocity of the cell fluid, $\Pi^{\to\to}$ is the viscous stress tensor, describing the inner friction in the cellular fluid, and$F_{\gamma}$is the external force acting on component γ of unit mass.

Non-equilibrium statistical physics affords an important clue for the understanding of the self-organization phenomena of living bodies. Prigogine proved that, in the linear range of an irreversible process in non-equilibrium thermodynamics, the entropy production always takes up a minimum if local equilibrium is assumed [6]. If the local equilibrium and its stability hold for each step of the process, minimum entropy production can be assumed not only for the linear region, but also for some non-linear regions, and in particular, for a system composed of chiral molecules such as the living body in the normal state (normal cells) [5,7].

In his famous book “What is life?”, SchrÖdinger pointed out that an organism feeds with negative entropy [1]. This means that entropy production in an organism is canceled by the outward entropy flow so that the system remains in a highly ordered state of low entropy. However, following our point of view, negative entropy (or negentropy) is only the first half of the story. The living organism is a chemical engine in which a series of chemical reactions take place one by one in an appropriate sequence. Accordingly, the energy transfer in an organism in the normal state is so efficient that the entropy production is minimized. Minimal entropy production in a healthy cell is the second half of the story [5].

**References**

1. SchrÖdinger E: *What is Life? Physical Aspects of Living Cell***.** University Press, Cambridge 1948, pp. 68-87.
2. Luo LF: **Information Biology – An Introduction.** *Acta Scientiarum Naturalium Universitatis Intramongolicae* 2005, **36:** 653-99.
3. Glansdorff P; Prigogine I: *Thermodynamic Theory of Structure, Stability and Fluctuations*. Wiley Interscience: New York, 1978, p 17.
4. Prigogine I: *Introduction to Thermodynamics of Irreversible Processes.* Interscience Publishers, John Willey, New York, 1967, pp 1-50.
5. Luo LF: *Theoretic-Physical Approach to Molecular Biology*. Shanghai Scientific and Technical Publisher 2004, pp. 572-578.
6. Nicolis BG, Prigogine I: *Self-organization in Nonequilibrium Systems*. Wiley Interscience: New York 1977. pp. 42-45.
7. Luo LF: **Comments on theorem of minimum entropy production and slaving principle.** *Acta Scientiarum Naturalium Universitatis Intramongolicae* 1993, **24:** 495-99. In: *Collected Works on Theoretical Biophysics*. Inner Mongolia University Press. 1997, p 471.
